# Familial MTC with *RET* exon 8 Gly533Cys mutation: origin and prevalence of second malignancy

**DOI:** 10.1530/EC-17-0147

**Published:** 2017-09-26

**Authors:** Katerina Saltiki, Elli Anagnostou, George Simeakis, Sofia Kouki, Anastasia Angelopoulou, Leda Sarika, Alexandra Papathoma, Maria Alevizaki

**Affiliations:** Endocrine UnitDepartment of Medical Therapeutics, National and Kapodistrian University of Athens, Alexandra Hospital, Athens, Greece

**Keywords:** RET, MTC, medullary thyroid cancer, exon 8, Gly533Cys, second malignancy, papillary coexistence, PTC

## Abstract

**Introduction:**

High prevalence of *RET* p.Gly533Cys (c.1597G > T) has been found in familial MTC in Greece (exon 8 fMTC). We studied their origin and compared clinical characteristics with non-exon 8 fMTC.

**Methods:**

102 fMTC (FMTC and MEN2A) patients (31.4% males) were followed for 2.9–37 years (median 6 years). Fifty-one carried the *RET* exon 8 mutation; the remaining were non-exon 8 fMTC (exons 10, 11, 13, 14). Pre-, post-operative calcitonin, disease extent at diagnosis and follow-up and families’ place of origin were recorded.

**Results:**

Exon 8 fMTC were older (42.3 ± 13.3 vs 30.8 ± 17.8 years, *P* < 0.001), including index cases (*P* = 0.016). In index cases, the stage at diagnosis was more favorable in exon 8 fMTC compared to non-exon 8 fMTC (stage I and II: 65% vs 23.8%, stage III: 25% vs 57.1%, stage IV: 10% vs 19%, *P* = 0.025). More favorable outcome was noted in exon 8 fMTCs (remission: 72.5% vs 45.8%, stable disease: 27.5% vs 41.7%, progression: 0.0% vs 12.5%, *P* = 0.001). Exon 8 fMTC patients carried more frequently a second malignancy (25.5% vs 6.3%, *P* = 0.009); 69% of these were PTCs. Exon 8 fMTC patients were significantly older at diagnosis compared to non-exon 8 moderate-risk *RET* carriers and presented more favorable clinical outcome (remission: 72.5% vs 50%, stable disease: 27.5% vs 41.7%, progression: 0.0% vs 8.3%, *P* = 0.021). This difference remained when only index cases were analyzed. ‘Hot spots’ in the origin of exon 8 fMTCs families were recognized. No phenotype or outcome differences were found between the exon 8 families from the various regions.

**Conclusions:**

In exon 8 fMTCs’ older age, favorable disease stage at diagnosis and favorable outcome suggest slow disease progression compared to non-exon 8 fMTC. Compared with moderate-risk *RET* mutation carriers, exon 8 fMTC patients have a more favorable clinical outcome. The higher prevalence of second malignancies, especially PTC, not previously reported, merits further investigation. Increased awareness for inherited disease is required for patients with apparently sporadic MTC originating from recognized ‘hot spots’, as the age at presentation is usually delayed.

## Introduction

Medullary thyroid carcinoma (MTC) accounts for 5–10% of all thyroid malignancies. About 25% of MTCs are familial (fMTC) and are described as multiple endocrine neoplasia syndromes: MEN2A (associated with pheochromocytoma and/or hyperparathyroidism), MEN2B (associated with marfanoid features and occasionally pheochromocytoma) and FMTC (familial medullary thyroid carcinoma only) which represents a variant along the spectrum of disease expression in MEN2A (1).

The disease is inherited in an autosomal dominant manner.

Mutations in the *RET* (rearranged during transfection) proto-oncogene are responsible for the transmission of fMTC. The *RET* oncogene is located on chromosome 10, consists of 21 exons and encodes a tyrosine kinase receptor involved in the growth and differentiation of neural crest-derived tissues (2). In 95% of the patients with typical MEN2A, *RET* mutations occur in codons 609, 611, 618 and 620 of exon 10 or in codon 634 of exon 11 (3). Uncommon mutations have been reported in the *RET* gene, such as codon 804 in exon 14, codon 883 in exon 15 and codons 515 and 533 in exon 8 (4, 5, 6). It appears that the spectrum of *RET* mutations in families with fMTCs varies in different countries (7, 8, 9).

The wide application of genetic testing has resulted in the recognition of previously undiagnosed hereditary disease in cases initially considered as sporadic (10). Thus, in recent years, the ‘rare’ exon 8 *RET* mutation p.Gly533Cys (c.1597G > T) has been found at high prevalence in patients with inherited MTC in Greece (11). Their significant proportion had previously been considered as sporadic, before the routine screening for this specific mutation had been introduced (11, 12).

A genotype–phenotype correlation in fMTC has been confirmed by several researchers. Particularly, concerning *RET* Gly533Cys exon 8 carriers, Signorini and coworkers reported a very comprehensive ten-year clinical update of 103 *RET* Gly533Cys carriers (12), while the ancestry related to the origin of G533C *RET* mutation in a large Brazilian kindred was recently studied (13). However, there are no detailed data concerning the geographical distribution of carriers of this specific *RET* mutation in Greece (11).

The aim of this study was to examine the phenotype and clinical outcome of *RET* exon 8 (Gly533Cys) carriers and compare these with non-exon 8 *RET* carriers with fMTC in Greece. Moreover, we aimed to record with more detail their place of origin and identify ‘hot spot’ areas in the country, so that a patient-centered and more individualized consultation to patients carrying this specific mutation could be provided.

## Patients and methods

One hundred two patients, diagnosed with familial MTC (FMTC and MEN2A), have been registered in the Endocrine Unit of the Academic Department of Clinical Therapeutics, during the last 37 years (mean follow-up 7.4 ± 5.5, median 6.0, range 2.9–37 years). Ninety-nine patients (30.3% males) carried a mutation in the *RET* gene and these were included in the analysis. In three patients with MTC, all members of one family, no RET mutation was identified and thus they were excluded from the analysis. For the purposes of this analysis, we excluded MEN2B patients as well as those *RET* carriers who had been identified through genetic screening and in whom histology had shown C cell hyperplasia only.

The genetic analysis was performed in genomic DNA extracted from peripheral blood lymphocytes. Genetic screening for *RET* mutation in exons 8, 10, 11, 13, 14 and 16 has been routinely performed from the year 2001 onward and retrospectively in all apparently sporadic MTC patients. The analysis for exon 15 mutations, which are rarely reported, was performed in patients diagnosed after 2005 and retrospectively in all apparently sporadic MTCs; no patient was found to carry this mutation in our cohort.

Fifty-one percent of the patients (*n* = 51) were *RET* Gly533Cys (exon 8 fMTC) carriers and the remaining 49% were *RET* non-exon 8 fMTCs (*n* = 48, exons 10, 11, 13, 14 – Table 1). Forty-one patients (41.4%) were index cases; the remaining were diagnosed after genetic screening. The study was conducted according to the Helsinki Declaration and was approved by the institutional ethical committee of ALEXANDRA Hospital review board, Medical School, Kapodistrian University of Athens. All patients except those lost to follow-up were informed about the purpose of the study and they gave their consent.

**Table 1:** Distribution of patients according to *RET* mutation.

**Exon**	**Number of patients**
8 (codon 533)	51
10 (codon 620)	19
11 (codon 634)	24
13 (codon 768)	3
14 (codon 804)	2
Total	99

The tumor size, the extent of the disease at diagnosis and during follow-up, the number of performed surgeries and the pre- and post-operative calcitonin levels were recorded. Staging at diagnosis was performed according to the American Joint Committee on Cancer (AJCC) TNM classification. The majority of surgeries were performed in 3 different collaborating Surgery Units by ‘high volume’ surgeons. Furthermore, detailed medical history for the presence of second malignancy was recorded.

Basal calcitonin and post-operative calcitonin at 3 and 6 months, and yearly after the first surgery were evaluated and used to classify patients into three groups: remission, stable disease and progressive disease. According to RECIST criteria and calcitonin doubling time, patients with normal post-operative calcitonin (<1.5 pg/mL) and negative imaging had ‘remission’, those with measurable post-operative calcitonin levels but without new imaging lesions had ‘stable disease’, while those with either calcitonin doubling time ≤1 year and/or new or increasing size lesions had ‘progressive disease’ (14).

Calcitonin screening has been routinely performed at our center since 2001 in patients with nodular thyroid disease. From 2006 to 2012, calcitonin was measured using a chemiluminescence DPC immunoassay (Immulite 2000, Siemens), while from 2000 to 2005, another chemiluminescence immunoassay was used (Nichols Institute Diagnostics, San Clemente, CA, USA). Before 2000, CIS Bio International ELISA-hCT Kit (IRMA, Cis-Diagnostics) was used. In patients in remission who were diagnosed before 2006, calcitonin was measured during follow-up with the sensitive method, confirming the very low calcitonin levels measured earlier. Patients with ‘biochemical cure’ were reevaluated with ultrasound imaging.

In addition, we recorded more detailed data concerning the place of origin of the exon 8 carriers, as well as that of their ancestors, up to 4–5 generations back. The patients belonged to 25 distinct families and were distributed in four age groups (G1–4) according to the age at diagnosis (age groups G1: ≤24 years, G2: 25–44 years, G3: 45–64 years and G4: ≥65 years).

### Statistical analysis

Statistical analysis was performed using the SPSS statistical package (version 18). All descriptive data are expressed as mean ± s.d. for normally distributed variables; otherwise, median value and interquartile range (IQR) are shown. The chi-square statistic and linear-by-linear association (Mantel Haenzel *χ*^2^) were used for contingency tables. For the comparison of the means, the *t*-test or the Mann–Whitney rank test was used depending on the normality of distribution. ANOVA or the Kruskal–Wallis test was used as appropriate. The Kaplan–Meier product limit method was used to estimate the probability of progression of disease 5 years (60 months) and 10 years (120 months) after initial diagnosis.

## Results

### Analyses in all fMTC patients (Table 2)

Exon 8 fMTC patients were significantly older at diagnosis compared to non-exon 8 *RET* carriers (42.3 ± 13.3 vs 30.8 ± 17.8 years, *P* < 0.001). This difference remained significant when only index cases were taken into account (48.7 ± 15.1 vs 44.9 ± 15.5, *P* = 0.016). No difference in sex distribution was observed. Exon 8 carriers had less frequent capsular infiltration (20.9% vs 42.9%, *P* = 0.037), soft tissue involvement (6.4% vs 19.4%, *P* = 0.07) and lymph node invasion (30.4% vs 57.9%, *P* = 0.01). No significant differences in multifocality, C cell hyperplasia and distant metastases at diagnosis were observed between groups. Tumor size did not differ either (Table 2).

**Table 2:** Characteristics of exon 8 fMTC and non-exon 8 fMTC patients (total sample and index cases).

	**Exon 8 fMTC**	**Non-exon 8 fMTC**		**Exon 8 fMTC**	**Non-exon 8 fMTC**	
**Patient characteristics**	Total sample (*n* = 51)	Total sample (*n* = 48)	***P*-Value**	Index cases (*n* = 20)	Index cases (*n* = 21)	***P*-Value**
Age	42.3 ± 13.3	30.8 ± 17.8	<0.001***	48.7 ± 15.1	44.9 ± 15.5	0.016***
Tumor size (cm) median (IQR)	0.8 (0.6)	1.5 (1.4)	0.17***	0.9 (1.0)	1.8 (1.8)	0.028***
Capsular infiltration	20.9%	42.9%	0.037*	25%	73.3%	0.007*
Lymph node invasion	30.4%	57.9%	0.01*	41.2%	82.4%	0.013*
Soft tissue involvement	6.4%	19.4%	0.07*	17.6%	28.6%	0.4*
Disease stage at diagnosis						
Stage I + II	70.6%	50%	0.042**	65%	23.8%	0.025**
Stage III	25.5%	41.7%		25%	57.1%	
Stage IV	3.9%	8.3%		10%	19%	
Pre-op CT (pg/mL) median (IQR)	80 (212)	225 (991)	0.9***	100 (208)	270 (8793)	0.174***
Post-op CT (pg/mL) median (IQR)	1.3 (9.5)	3.1 (39)	0.2***	4.2 (9.5)	6 (12.1)	0.25***
Outcome						
Remission	72.5%	45.8%	0.001**	65%	23.8%	0.002**
Stable	27.5%	41.7%		35%	52.4%	
Progression	0.0%	12.5%		0.0%	23.8%	

* Pearson’s *χ* ^2^ test, **linear-by-linear association test, ***Mann–Whitney test.

IQR, interquartile range.

A significant difference in the disease stage at diagnosis was observed between the 2 groups (*RET* exon 8 vs non-exon 8 fMTC: stage I + II: 70.6% vs 50%, stage III: 25.5% vs 41.7%, stage IV: 3.9% vs 8.3%, *P* = 0.042, linear-by-linear association). Moreover, when patients diagnosed after genetic screening were excluded from the analysis (only index cases, *n* = 41), the stage at diagnosis was significantly more favorable in *RET* exon 8 fMTC compared to non-exon 8 fMTC (stage I + II: 65.0% vs 23.8%, stage III: 25.0% vs 57.1%, stage IV: 10.0% vs 19%, *P* = 0.025, linear-by-linear association). Pre-operative and post-operative calcitonin levels did not differ between exon 8 and non-exon 8 carriers (Table 2). There was no difference in the type of the first surgery.

**Figure 1:**
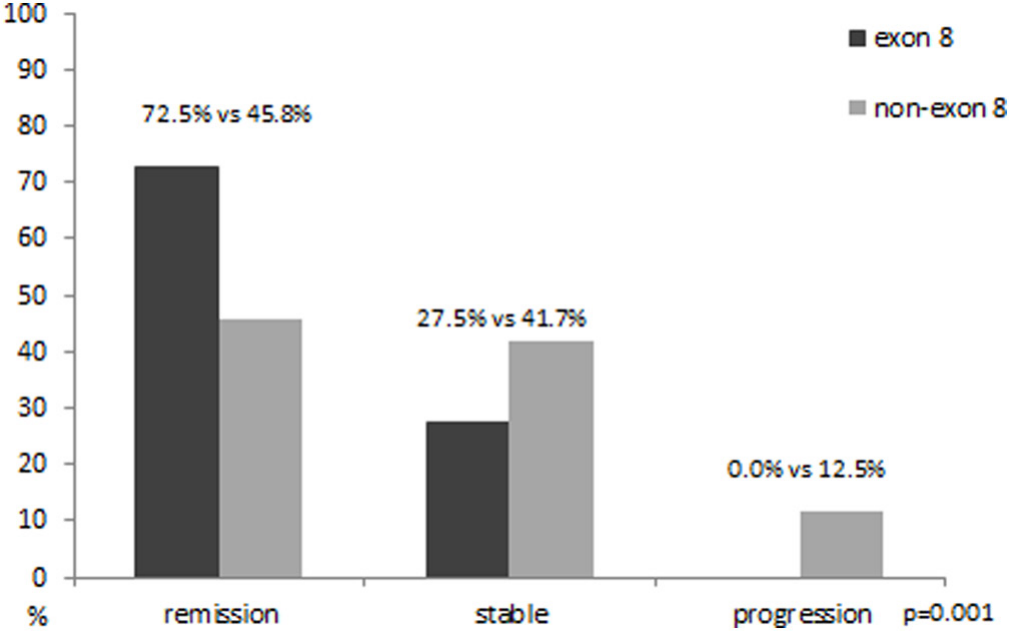
Disease outcome in the two fMTC groups, *RET* exon 8 and *RET* non-exon 8 carriers, in the entire case series of fMTC.

**Figure 2:**
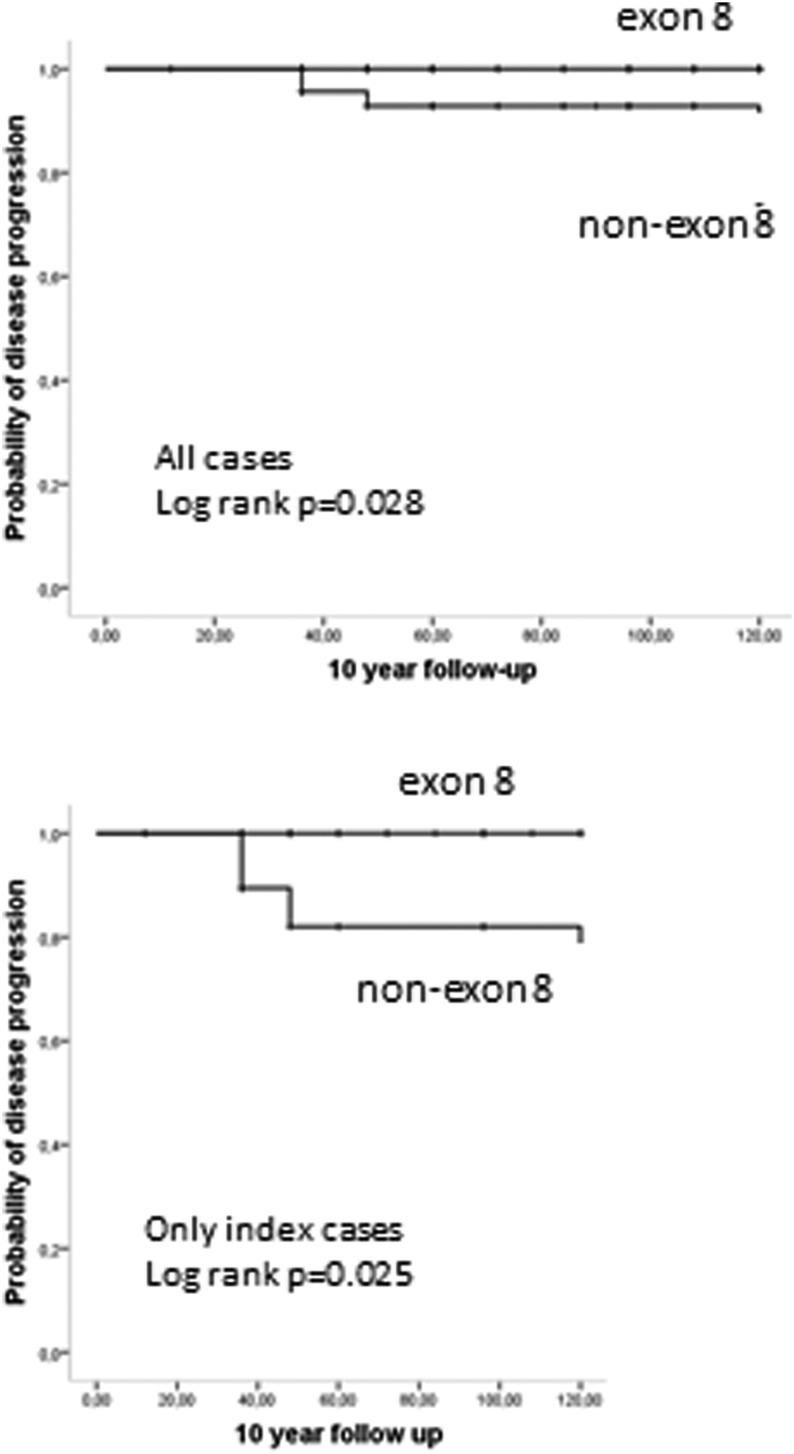
10-year probability of lack of progression of disease according to the *RET* mutation (exon 8 and non-exon 8 carriers) in the entire case series and in index cases (after excluding those diagnosed through genetic screening).

Significantly more favorable clinical outcome was noted in the group of *RET* exon 8 fMTC compared to non-exon 8 fMTC (remission: 72.5% vs 45.8%, stable disease: 27.5% vs 41.7%, progression: 0.0% vs 12.5%, *P* = 0.001, linear-by-linear association, Fig. 1). Similar results were obtained when only index cases were analyzed (*P* = 0.002). The 5-year probability of lack of progression of disease differed significantly when *RET* exon 8 and non-exon 8 fMTC patients were compared (100% vs 87.5%, Kaplan–Meier analysis, *χ*^2^ = 4.36, *P* = 0.012 for log rank); the same was noticed concerning the 10-year probability of lack of progression of disease (100% vs 87.8%, Kaplan–Meier analysis, *χ*^2^ = 4.82, *P* = 0.028 for log rank, Fig. 2). Similar results were obtained when only index cases were included in the analysis (5-year probability: 100% vs 76.2%, Kaplan–Meier analysis, *χ*^2^ = 6.46, *P* = 0.011 for log rank, 10-year probability: 100% vs 76.2%, Kaplan–Meier analysis, *χ*^2^ = 5.03, *P* = 0.025 for log rank, Fig. 2).

Interestingly, a higher percentage of *RET* exon-8 fMTC patients carried a second malignancy either at diagnosis or at follow-up (25.5% vs 6.3%, *P* = 0.009, Pearson’s *χ*^2^): of the *RET* exon 8 carriers, 9/51 (17.6%) had papillary thyroid cancer (PTC) and another 4/51 (7.8%) had other malignancies (lung, renal, unknown primary tumor, breast); thus, in 69% of the cases, the second malignancy was PTC. The PTC diagnosis in all patients was simultaneous to the MTC diagnosis, during the first surgery. These PTC tumors were in their majority micro-PTC (7/9), while 3/9 were multifocal. The mean age at diagnosis of these patients was 45.2 ± 13.4 years (median 45 years). The majority had typical PTC features, while 1/9 was classified as tall cell variant. Lymph node metastases from PTC were present in 2/9 patients (the tall cell variant and one classical PTC). Three out of 9 patients received Radioactive Iodine therapy. All patients are in remission concerning PTC. Of the non-exon 8 carriers, only 1/48 (2.1%) had papillary thyroid cancer (micro-PTC) and another 2/48 (4.2%) had a second neoplasia (breast, sarcoma). There was a statistically significant difference in the presence of PTC between exon 8 carriers and non-exon 8 carriers (17.6% vs 2.1%, *P* = 0.01, Pearson’s *χ*^2^).

### Analysis of moderate-risk fMTC patients

Furthermore, we performed an analysis excluding the High-Risk RET mutation carriers according to ATA risk classification, that is, all exon 11 (codon 634) patients. Thus, this analysis was performed between exon 8 fMTC patients (*n* = 51) and non-exon 8 moderate-risk *RET* mutation carriers (*n* = 24).

Exon 8 fMTC patients were significantly older at diagnosis compared to non-exon 8 moderate-risk *RET* carriers (42.3 ± 13.3 vs 35.8 ± 29 years, *P* < 0.02). This difference was not significant when only index cases (*n* = 20 and *n* = 9) were taken into account. No difference in capsular infiltration, soft tissue involvement, lymph node invasion and tumor size was observed. No statistically significant difference in the disease stage at diagnosis was observed between the 2 groups.

Nevertheless, significantly more favorable clinical outcome was noted in the group of *RET* exon 8 fMTC compared to non-exon 8 moderate-risk RET group (remission: 72.5% vs 50%, stable disease: 27.5% vs 41.7%, progression: 0.0% vs 8.3%, *P* = 0.021, linear-by-linear association). Similar results were obtained when only index cases were analyzed (remission: 65% vs 22.2%, stable disease: 35% vs 66.7%, progression: 0.0% vs 11.1%, *P* = 0.019, *P* = 0.002, linear-by-linear association).

### Subgroup analysis of *RET* exon 8 carriers

Subsequently, we analyzed the clinical and laboratory parameters in the group of *RET* Gly533Cys carriers. Mean age at diagnosis in the four age subgroups (G1–4) was as follows: 21.0 ± 2.9 (youngest age group G1, *n* = 4), 35.8 ± 4.9 (G2: 24–44 years, *n* = 28), 52.7 ± 6.0 (G3: 45–64 years, *n* = 14) and 68.4 ± 3.7 (oldest, G4: ≥65 years, *n* = 5). Twenty patients were index cases diagnosed at our center. Twelve belonged to G2, four to G3 and four to G4.

**Figure 3:**
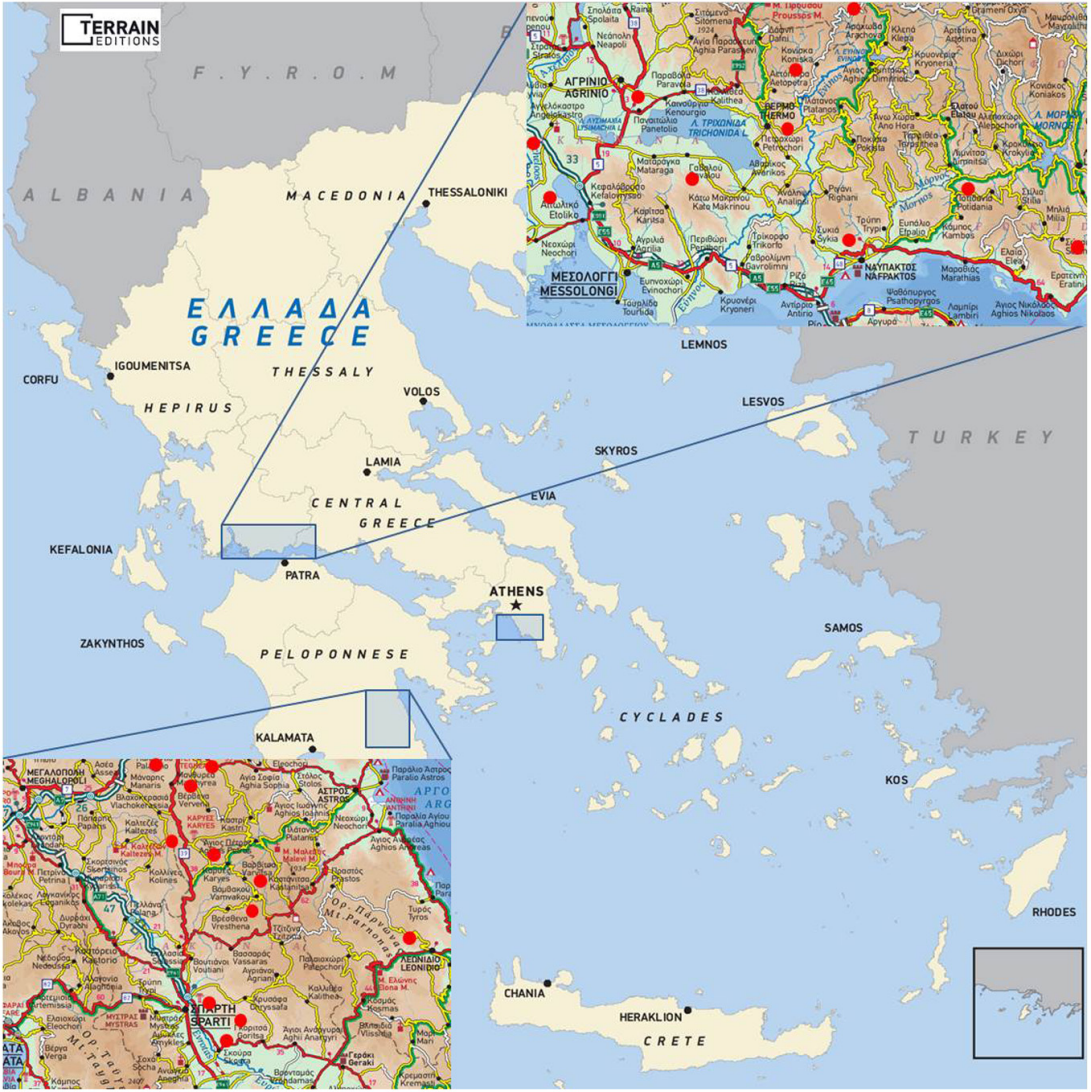
Geographical distribution of RET exon 8 carriers in Greece indicating ‘hot spot’ areas of origin of *RET* exon 8 carriers in Central/Western Greece (around Lake Trichonis, and Fokis) and in Peloponnese (Laconia and Arcadia, mount Parnon region). Reproduced, with permission, from TERRAIN Editions publishers.

The patients belonged to 25 families. ‘Hot spots’ for the origin of these families were recognized (Fig. 3). Ten families originated from Central/Western Greece in an area around Lake Trichonis and Fokis, eleven originated from Peloponnese (Laconia (mount Parnon region) and Arcadia), three from the Attika region and one family from Asia Minor (Smyrna region), all of them without any recognized family relationship (Fig. 3). Other three of the aforementioned families reported a distant ancestry from the same region in Asia Minor. No phenotype or outcome differences were found between the families from the various regions. Only one patient had pheochromocytoma; no patient had hyperparathyroidism.

## Discussion

Our cohort of fMTCs is enriched in exon 8 carriers. Indeed, the exon 8 Gly533Cys mutation appears to be commonly detected in Greece (11, 15, 16, 17) and the Mediterranean region (6). Da Silva and coworkers were the first to report this mutation in a six-generation family from Brazil, with ascendants from Spain. Unpublished data report at least one Gly533Cys family in South Portugal and a few families in the French registry (reported in (8)). One patient of Slovenian origin has been reported in Ireland (18). Interestingly, the first family identified in the United States carrying this mutation was of Greek ancestral origin (19). Mutations other than codon 533 in the exon 8 of the *RET* gene have occasionally been reported in the literature (5, 20, 21, 22); however, none of them has been found in families of Greek origin.

As *RET* exon 8 carriers (Gly533Cys) are quite prevalent in Greece, we aimed to clarify whether fMTC patients carrying this mutation present different clinical features compared with fMTC carriers of other *RET* mutations. Several findings in our study point to a mild phenotype in fMTC patient carriers of this mutation. One finding was that *RET* exon 8 fMTC patients had significantly higher age at diagnosis, a difference that remained significant when patients diagnosed after genetic screening were excluded. In a study where patients carrying *RET* proto-oncogene mutations considered ‘Low Risk’ by ATA were examined, it was found that for codons 533, 609, 611, 791 and 804, the appearance of MTC before age 20 was ≤10%, while for codon 533, it was 63% by age 50; the estimated median age at diagnosis was 47 years (23). Our findings agree with these data.

Accordingly, the stage of disease at diagnosis and the clinical outcome were more favorable for the exon 8 fMTC patients compared to non-exon 8 fMTCs. Similar results were obtained when only index cases were analyzed, supporting the aforementioned data. Moreover, when exon 8 fMTC patients were compared to non-exon 8 moderate-risk *RET* patients, no difference concerning the stage of disease at diagnosis was identified; however, the clinical outcome was more favorable for the exon 8 fMTC patients compared to non-exon 8 moderate risk *RET* patients. The study of Signorini and coworkers reporting a ten-year clinical update of a large *RET* Gly533Cys fMTC kindred indicates a large clinical variability, ranging from only elevated calcitonin level (3%) to local metastatic disease (25%); 42% of the individuals were cured and the majority (56%) had stable chronic disease (12). The later appearance and the milder course of the disease in our series agree with these findings and support the favorable clinical features found in *RET* exon 8 fMTC carriers in general. Taken together, these findings confirm that *RET* exon 8 mutation fMTC is indeed a medium-risk thyroid cancer as also suggested by the recently revised ATA guidelines (1).

An interesting finding was the higher prevalence of a second malignancy either at diagnosis or at follow-up in *RET* exon 8 fMTC patients compared to *RET* non-exon 8 fMTCs. The co-occurrence of MTC and PTC in hereditary MTC patients is interesting and has not been described before in G533C carriers. Regarding MTCs, a coexistence of a variety of other malignancies has occasionally been reported (24, 25, 26, 27, 28). However, specifically for familial cases, few reports have examined the coexistence of primary malignancies other than those included in the MEN syndromes. Signorini and coworkers in the long-term follow-up of 533 carriers reported cases of melanoma, lung and breast cancer (12). Gagel and coworkers reported two patients with fMTC that died from another primary malignancy in a large family with MEN2a (29). In our cohort, the most frequent second malignancy in *RET* exon 8 carriers was papillary thyroid carcinoma (PTC), accounting for 69% of the cases of second malignancy. Concerning this coexistence, common pathogenetic mechanisms have been suggested (30, 31, 32, 33). It is known that the *RET* gene is involved in the sporadic form of both these thyroid malignancies: in sporadic MTC as somatic mutation, and in PTC as somatic mutation/rearrangement. Concerning hereditary MTC, Machens and Dralle reported that 5/6 MEN2A patients with simultaneous MTC and PTC carried a relatively late discovered non-cysteine RET mutation; one L790F carrier; two V804L and 2 S891A carriers (34). Schulte and coworkers described this co-occurrence in 4/29 (14%) S891A carriers and Shifrin and coworkers in 6/15 (40%) V804M carriers (35, 36). In some familial cases where the two cancers coexist, polymorphisms in the *RET* gene have been speculated to act as ‘modifiers’ of *RET* expression (37). However, no associations with any germ line *RET* mutation have been reported. In a recent study by Ciampi and coworkers, where mainly sporadic MTCs were examined, no common molecular defects were identified between PTCs and coexisting MTCs (38). Thus, as far as we know, there is no known etiologic factor that can explain a second malignancy such as PTC in these patients. As such a finding has not been previously reported, it merits further investigation. One cannot exclude the possibility that these PTCs were only diagnosed because the patient underwent thyroidectomy; however, this consideration also applies to the non-exon 8 fMTCs.

In our study, we further investigated the places of origin of the different 25 families of exon 8 carriers. It is interesting that we recognized several ‘hot spot’ areas in Greece. The majority of patients originate from Central/Western Greece (in an area around Lake Trichonis and in Fokis) and Peloponnese (around mount Parnon region and in Arcadia), all of them without any recognized familial relationship. Further ‘hot spots’ were the Attika region and the distant ancestry from Asia Minor. The hypothesis that Spanish/Brazilian exon 8 carriers might share a common ancestral origin with Greek exon 8 carriers was recently tested by our group in a collaborative study. The results indicate that patients from our series share common haplotypes with those of the large Spanish/Brazilian pedigree (13). In that study, we included patients from eight distinct Greek families from different ‘hot spot’ regions. The results showed that these too share common haplotypes between them indicating that, as expected from this clustering, they have a common unrecognized ancestor (13).

We can therefore support that increased awareness for inherited disease is required for patients with apparently sporadic MTC originating from these areas, as the age at presentation is usually delayed (≥25 years).

## Conclusions

Familial MTC due to exon 8 *RET* mutation is frequently diagnosed in recent years in the Greek population. The age at diagnosis is higher in *RET* exon 8 fMTC carriers compared to non-exon 8 fMTC. The outcome of the disease is more favorable suggesting relatively slow disease progression. The higher prevalence of second malignancies, especially PTC, has not been previously reported and merits further investigation.

Concerning *RET* exon 8 (Gly533Cys) carriers, the majority of them originate from Central/Western Greece and Peloponnese. Increased awareness for inherited disease is required for patients with apparently sporadic MTC originating from these areas, as the age at presentation is usually delayed.

## Declaration of interest

The authors declare that there is no conflict of interest that could be perceived as prejudicing the impartiality of the research reported.

## Funding

This research did not receive any specific grant from any funding agency in the public, commercial or not-for-profit sector.

## Author contribution statement

All the authors have made a significant contribution to the findings and methods in the paper. Namely, K S drafted the manuscript. E A along with G S reviewed the literature. S K and A A extracted the clinical data. L D and A P carried out the genetic screening. M A participated in the design and coordination of the task and helped to draft the manuscript. All authors read and approved the final manuscript.
